# Combined Influences of Water Content and Coarse Grain Content on Shear Strength of Unsaturated Soil Mixture

**DOI:** 10.3390/ma16206657

**Published:** 2023-10-11

**Authors:** Yu Su, Bo Han, Junyi Duan, Fumin Zhao

**Affiliations:** 1 Jiangxi Hydraulic Safety Engineering Technology Research Center, Jiangxi Academy of Water Science and Engineering, Nanchang 330029, China; yusu@ncu.edu.cn; 2 School of Infrastructure Engineering, Nanchang University, Nanchang 330031, China; 412500220006@email.ncu.edu.cn (B.H.); xyzfm@126.com (F.Z.)

**Keywords:** fine/coarse soil mixture, coarse grain content, shear strength, suction, constitutive model

## Abstract

An interlayer existed between the ballast layer and subgrade in the conventional railway substructure. Considering that the shear strength *τ* of the interlayer soil was influenced by the changes in the ballast grain content and water content, this aspect was explored in the present study. Monotonic triaxial tests were fulfilled, which considered five coarse grain contents *f*_v_ and three water contents of fine soil *w*_f_. The results showed that the growth in *f*_v_ contributed to an increment in *τ* of the soil mixture under both saturation and unsaturation. Conversely, in previous studies, the growth of *f*_v_ induced an increment in *τ* under saturation, but a decline in that under unsaturation. This was explained by the competing influences of *f*_v_ and suction *ψ*: in previous studies, increasing *f*_v_ induced a decline in the dry density of the fine soil fraction *ρ*_d–f_, which contributed to a decline in *ψ*. When the negative influence of declining *ψ* outweighed the positive influence of the incrementing *f*_v_, the *τ* of the soil mixture decreased. Meanwhile, modelling of the *τ*–*ψ* relationship in the soil mixture with varying *f*_v_ was performed. This proposed model was examined using the test results from both the present and previous studies, which shows its reasonably good performance.

## 1. Introduction

Over the years of trains moving loads, interpenetration between the ballast layer and subgrade occurred, leading to the formation of an interlayer in the conventional railway track (Trinh [1]). This interlayer soil plays an important role for the railway track in (a) spreading the traffic loadings from the ballast layer to the subgrade to avoid failure because of excessive deformation, and (b) eliminating the effect of rainwater seepage in the subgrade (Trinh et al. [2]). Field observation shows a decline in ballast grain content with the incrementing depth of the interlayer. Due to the unstable water content in the field, the water content of the interlayer soil varied frequently. In this circumstance, the shear strength *τ* of the interlayer soil was significantly affected by the changes in the ballast grain content and water content, which was of great importance for the stability and safety of the railway track. To ensure the good performance of railway track, it appears important to study this aspect in depth.

Numerous studies have examined the influence of water content on *τ* of the soil mixture (Duong et al. [3]; Qi et al. [4,5]; Wan et al. [6]; Bian et al. [7]). In the saturated state, the pore water pressure was accumulated under the influence of train moving loads, resulting in a decline in *τ*. With declining water content, the shear strength *τ* increased owing to an increase in suction. The influence of coarse grain content on the shear strength *τ* of the mixture was explored in previous studies. Vallejo et al. [8] explored the *τ* of a fine/coarse soil mixture using two glass beads, with a size of 0.4 and 5 mm. They found that increasing the coarse grain content induced a transition in the soil structure from a fine-grain-supported structure to a coarse-grain-supported structure. Seif EI Dine et al. [9] carried out triaxial tests to explore the *τ* of a mixture with a gravel and sandy soil matrix. From these tests, an increment in the gravel content from 0% to 35% contributed to a small increment in the *τ* of the mixture. That is because in this range of gravel content, the *τ* of the soil mixture was mainly dominated by the sandy matrix. Wang et al. [10] explored the *τ* of a soil mixture with a wide range of coarse grain content *f*_v_ (defined by the volumetric proportion of the coarse grains to the mixture), from 0% to 45%. They found a characteristic *f*_v-cha_ value: *τ* incremented with the incrementing *f*_v_ slightly when *f*_v_ < *f*_v-cha_, but largely when *f*_v_ > *f*_v-cha_. Correspondingly, two fabrics in the mixture were identified: a fine-grain-supported fabric when the *f*_v_ < *f*_v-cha_ and a coarse-grain-supported fabric when *f*_v_ > *f*_v-cha_. It is noted that the influence of the water content *w* on the *τ* of the mixture with these two fabrics was not investigated by Wang et al. [10]. Duong et al. [3] explored the influence of *w* and *f*_v_ on the *τ* of the interlayer soil with varying *f*_v_ = 50~56% using large-scale triaxial tests. From these tests, the coupling effects of *w* and *f*_v_ on the *τ* were observed: increasing *f*_v_ resulted in an increment in *τ* at saturation, while a reverse trend was obtained during unsaturation. One should pay attention to the fact that the interlayer soil with *f*_v_ = 50~56% only has the coarse-grain-supported fabric, without the fine-soil-supported fabric. Up to now, the influences of *w* and *f*_v_ on the *τ* of a soil mixture with two varying fabrics have not been investigated yet.

Different models were proposed for describing the *τ* of unsaturated soils. Abramento and Carvalho [11] investigated the *τ* and suction *ψ* of residual soil from natural slopes, and developed an exponential function between *τ* and *ψ*ψ. Vanapalli et al. [12] predicted the *τ* of unsaturated soil as a function of suction *ψ* using the soil water retention curve (SWRC), which was verified by the experimental results on a glacial till. Vilar [13] proposed an empirical hyperbolic formulation for describing the *τ*–*ψ* relationship, with one set of measured experimental data required for its application. Han and Vanapalli [14] developed a normalized function to describe the *τ*–*ψ* relationship, which incorporated the SWRC. This model was successfully applied to a given soil, such as coarse-grained sands and expansive clays. However, no model describes the *τ*–*ψ* relationship for a soil mixture with varying *f*_v_ values.

This study explored the combined influence of *w* and *f*_v_ on the *τ* of a soil mixture. Monotonic triaxial tests were fulfilled, which considered the *f*_v_ and water content of fine soil *w*_f_ values. The experimental results show the variation in the *τ* of a soil mixture according to the *f*_v_ and *w*_f_. Afterwards, modelling of the shear strength *τ* of the soil mixture was performed by incorporating the SWRC, which was examined using both the test results from the present study and those from previous studies.

## 2. Materials and Methods

### 2.1. Reconstituted Fine Soil and Micro-Ballast

**Figure 1:**
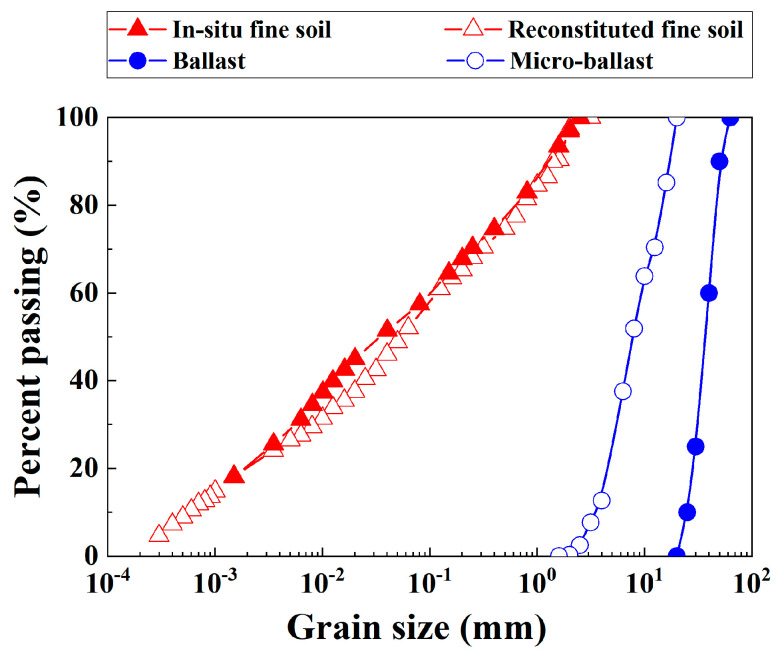
Grain size distribution curves for fine soil and coarse grains.

The interlayer soil was substituted by a mixture of reconstituted fine soil and micro-ballast in the laboratory study. Figure 1 presents a comparison of the grain size distribution (GSD) curves between in situ fine soil and reconstituted fine soil, which shows good agreement.

The basis soil properties of the reconstituted fine soil are as follows: the specific gravity *G*_s_ = 2.68, the liquid limit *w*_L_ = 32%, the plasticity index *I*_p_ = 20%, the optimum water content of the fine soil *w*_opt-f_ = 13.7% and the maximum dry density of the fine soil *ρ*_dmax-f_ = 1.82 Mg/m^3^, respectively.

The GSD curve for the micro-ballast was obtained from that of ballast based on the parallel similitude method (Figure 1), which was consistent with Wang et al. [10]. In accordance with Wang et al. [10,15], the parameter *f*_v_ was employed for quantifying the volume of coarse grains in the soil mixture.

Five *f*_v_ values (0%, 10%, 20%, 35% and 45%) and three *w*_f_ values (7.0%, 10.6% and 17.6%) were considered. Note that *w*_f_ = 7.0%, 10.6% and 17.6% corresponded to the degree of saturation *S*_r_ = 40%, 60% and 100%, respectively. The mixture of reconstituted fine soil and micro-ballast with varying *f*_v_ values was compacted to attain a cylindrical sample (diameter *d* = 100 mm, height *h* = 200 mm).

**Figure 2:**
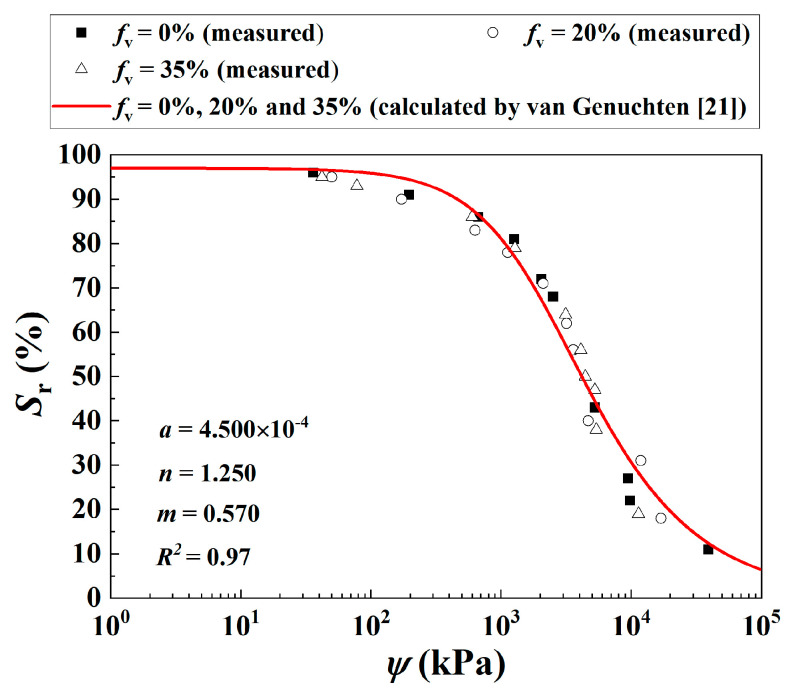
Measured and calculated SWRC for the soil mixture with different *f*_v_ values (data from Su et al. [20]).

Note that during the compaction, the fine soil fraction was controlled at a constant *w*_opt-f_ = 13.7% and *ρ*_dmax-f_ = 1.82 Mg/m^3^. As a result, the target dry density of the mixture *ρ*_d_ increased with increasing *f*_v_. The as-compacted samples with a constant *w*_opt-f_ = 13.7% and varying *f*_v_ values were then either dried to *w*_1-f_ = 7.0% and *w*_2-f_ = 10.6% or wetted to *w*_3-f_ = 17.6%, with reference to the protocols developed by Su et al. [16] and Han and Vanapalli [17]. In the drying procedure, 1 h of air drying was adopted each time, followed by an equilibration time of at least 7 h, which minimizes the development of cracks and fissures. In the wetting procedure, an increment of 10 g water was employed each time, with the same equilibration time adopted. The drying/wetting procedure adopted was based on the consideration that after compaction at optimum water content, the in situ soil mixture was dried to a lower or wetted to a higher water content and attained an equilibrium state with the external environment (Yang et al. [18,19]). Figure 2 depicts a unique soil water retention curve (SWRC) for a mixture with an unchanged *ρ*_dmax-f_ = 1.82 Mg/m^3^ and different *f*_v_ = 0%, 20% and 35% (Su et al. [20]).

The SWRC for the mixture was described by the van Genuchten [21] model with the parameters *a* = 4.500 × 10^−4^, *n* = 1.250, *m* = 0.570. Note that the parameter *a* approximates the inverse of the air-entry pressure, the parameter *n* related to the pore size distribution of the soil and the parameter *m* controlled the symmetry of the soil water retention curve. More details on the water retention properties of the mixture can be found in Su et al. [20].

### 2.2. Monotonic Triaxial Tests

Monotonic triaxial tests were fulfilled for the determination of the shear strength *τ* of the mixture with five *f*_v_ values (0%, 10%, 20%, 35% and 45%) and three *w*_f_ values (7.0%, 10.6% and 17.6%). The values of the confining pressure *σ*_3_ were 30, 60 and 120 kPa based on the consideration of the traffic loadings and the interlayer soil’s depth, which were consistent with those in Wang et al. [10]. In the case of *w*_f_ = 17.6% (*S*_r_ = 100%), the confining pressure *σ*_3_ was applied overnight, allowing the dissipation of the pore water pressure. This was followed by the shearing process until the end of the tests. In the case of *w*_f_ = 7.0% and 10.6% (*S*_r_ = 40% and 60%), the same consolidation time overnight was adopted prior to shearing. All the tests were fulfilled with a low shear rate of 0.1 mm/min. These tests were performed with reference to the protocol adopted by Wang et al. [10]. The test ended with either a peak deviator stress that presented when the axial strain *ε*_a_ < 15%, or when the axial strain *ε*_a_ = 15% (ASTM D7181-11 [22]).

## 3. Experimental Results

**Figure 3:**
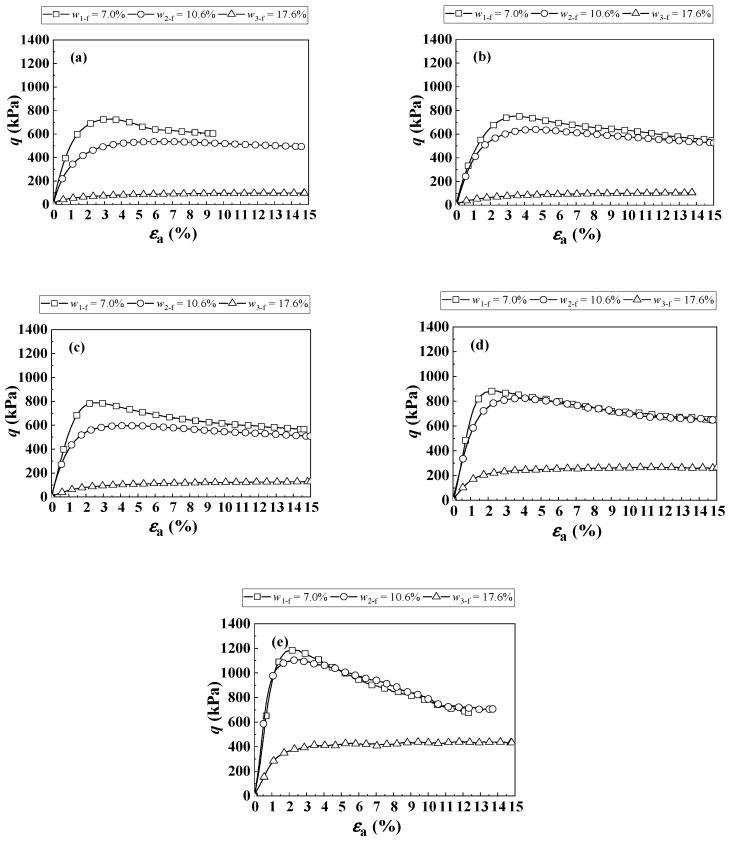
Shear behaviors of the soil mixture under a constant $\sigma_{3}$ = 120 kPa: (**a**) *f*_v_ = 0%, (**b**) *f*_v_ = 10%, (**c**) *f*_v_ = 20%, (**d**) *f*_v_ = 35% and (**e**) *f*_v_ = 45%.

Figure 3 shows the shear behaviors of the soil mixture with different *f*_v_ and *w*_f_ values under a constant *σ*_3_ = 120 kPa.

For the deviator stress *q*-axial strain *ε*_a_ curves at *f*_v_ = 0% (Figure 3a), under a given *w*_f_, the *q* increased with an increment in the *ε*_a_ until a peak value, prior to its decline. With increasing *w*_f_, the *q* decreased significantly. This could be attributed to the decrease in suction *ψ*. Similar observations can be made when *f*_v_ = 10–45% (Figure 3b–e). It was found that under a constant *w*_f_, no matter whether the conditions were saturated and unsaturated, an increase in *f*_v_ from 0% to 45% led to an increase in *q* (Figure 3a–e), which was due to the reinforcement effect of coarse grains. A similar observation was made by Wang et al. [10]. 

**Figure 4:**
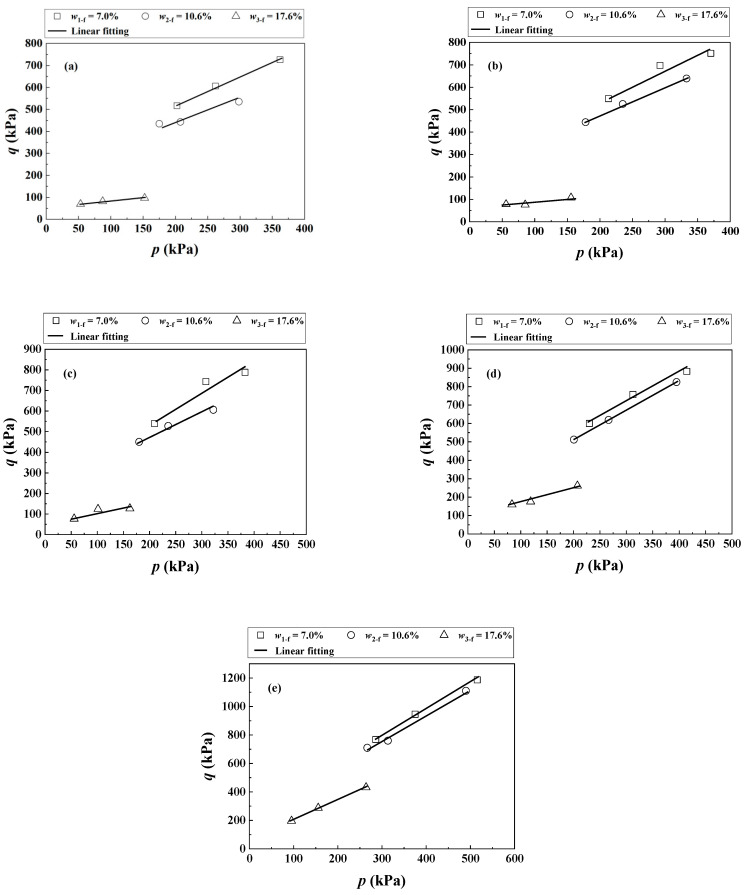
Failure envelopes of the soil mixture under (**a**) *f*_v_ = 0%, (**b**) *f*_v_ = 10%, (**c**) *f*_v_ = 20%, (**d**) *f*_v_ = 35% and (**e**) *f*_v_ = 45% in the *q*-*p* plane.

For further analysis, the deviator stress *q* at failure was selected, which was defined as the peak deviator stress or the deviator stress at *ε*_a_ = 15% (ASTM D7181-11 [22]). Figure 4 shows the failure envelops of the soil mixture with different *f*_v_ = 0%, 10%, 20%, 35% and 45% in the *q-p* plane, where *p* is the mean stress.

At *f*_v_ = 0% (Figure 4a), an increase in the *w*_f_ induced a decrease in the slope and intercept of the failure envelops. Similar observations were made for *f*_v_ = 10~45% (Figure 4b–e). Correspondingly, the cohesion *c* and friction angle *φ* of the soil mixture with different *f*_v_ and *w*_f_ were determined, as conducted by Trinh et al. [2] and Wang et al. [10].

Table 1 presents the cohesion *c* and friction angle *φ* of the soil mixture with varying *w*_f_ and *f*_v_ values. It can be observed that under a given *w*_f_, increasing the *f*_v_ induced a decline in *c*, which was owing to the decrease in the fine soil content. With an increase in *w*_f_, the *c* decreased due to the decrease in suction. It was found that under a constant *w*_f_, increasing the *f*_v_ resulted in an increase in *φ*, due to more coarse grains involved during the shearing. When *w*_f_ decreased, the *φ* was observed to increase. That could be explained by the suction-induced aggregation of the fine soil: according to the findings by Delage et al. [23], a fine matrix fabric was formed on the wet side of the optimum (e.g., *w*_3-f_ = 17.6% > *w*_opt-f_ = 13.7%), while a fine aggregate fabric was obtained on the dry side (e.g., *w*_1-f_ = 7.0% and *w*_2-f_ = 10.6% < *w*_opt-f_ = 13.7%). The decrease in *w*_f_ from 17.6% to 10.6% and 7.0% contributed to a change in the fabric of the fine soil fraction from a fine matrix fabric to a fine aggregate fabric. This change in the fabric resulted in an increase in *φ*. Similarly, suction-induced aggregation of unsaturated loess was observed by Ng et al. [24] using scanning electron microscopy; who reported that an increase in suction up to 40 MPa gave rise to a notable increase in soil stiffness.

**Figure 5:**
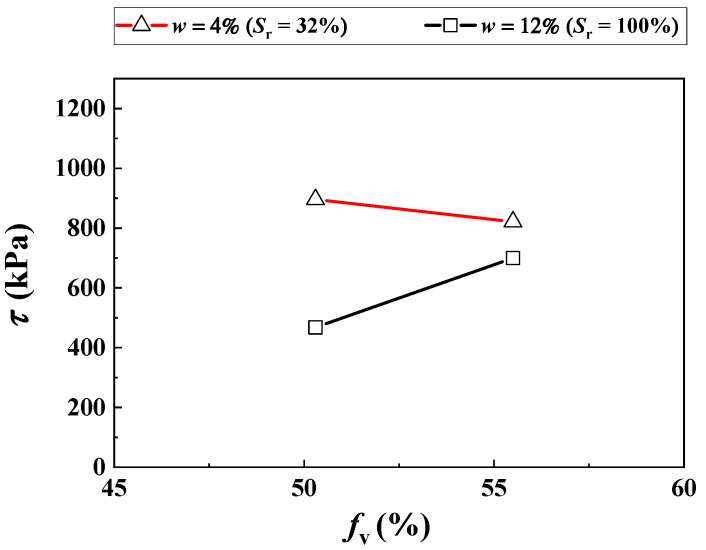
Variations in the shear strength with *f*_v_ under different water contents (after Duong et al. [3]).

Figure 5 shows the shear strength *τ* of the soil mixture with various *f*_v_ = 50% and 56% and a water content of mixture *w* = 4% and 12%, which were obtained from Duong et al. [3].

It is noted that in situ fine soil and ballast were employed by Duong et al. [3], which were different from the reconstituted fine soil and micro-ballast in the present study (Figure 1). For the fine soil fraction, the GSD curve for the reconstituted fine soil was consistent with that of the in situ fine soil (Figure 1). For the coarse grain fraction, according to the findings of Qi et al. [5], micro-ballast can be adopted as a substitute for the ballast when studying the mechanical behaviors of interlayer soil. Figure 5 shows that with increasing *f*_v_, the *τ* decreased under the unsaturated condition (*w* = 4%, *S*_r_ = 32%) but increased under the saturated condition (*w* = 12%, *S*_r_ = 100%). These observations were different from those in the present study, which found that the increasing *f*_v_ induced an increase in the shear strength *τ* under both unsaturated and saturated conditions. Note that the *τ* was taken as the deviator stress at failure in Figure 4. This was explained as follows: in Duong et al. [3], a stable dry density of the mixture *ρ*_d_ = 2.01 Mg/m^3^ was adopted. In this case, the growth of *f*_v_ from 50% to 56% induced a decline in the dry density of the fine soil fraction *ρ*_d–f_ from 1.33 to 1.17 Mg/m^3^, and hence a decline in suction *ψ*.

While the negative influence of the declining *ψ* outweighed the positive influence of the incrementing *f*_v_, the *τ* was observed to decrease. On the contrary, a stable *ρ*_d–f_ = 1.82 Mg/m^3^ was adopted in the present study. This gave rise to a constant suction ψ in the mixture with increasing *f*_v_, as shown in Figure 2 (Su et al. [20]). In this case, the increasing *f*_v_ contributed to an increase in the *τ* of the soil mixture under both saturation and unsaturation, due to its only positive reinforcement influence.

Comparisons between Duong et al. [3] and the present study show that the *τ* of the soil mixture was notably influenced by the *f*_v_, *w* and *ρ*_d–f_. The combined influence of the *w* and *ρ*_d–f_ on the *τ* could be reflected by the influence of the *ψ*. The SWRC was incorporated into different models to describe the effect of the *ψ* on the *τ*, such as the models by Vanapalli et al. [12] on glacial till and Han and Vanapalli [14] on cohesionless or cohesive soil. To date, no model describes the *τ–ψ* relationship of the soil mixture with different *f*_v_ by incorporating the SWRC.

## 4. Modelling of Shear Strength

Referencing the existing models (Equations (1)–(6) from Abramento and Carvalho [11], Vanapalli et al. [12], Öberg and Sallfors [25], Xu and Sun [26], Vilar [13] and Han and Vanapalli [14], repectively in Table 2), a general form of unsaturated shear strength *τ* was obtained, as shown in Equation (7):

**Table 2:** Shear strength equations for unsaturated soils in previous studies.

Reference	Equation	
Abramento and Carvalho [11]	$\tau =c^{\prime }+\left(\sigma -u_{a}\right)\tan\varphi^{\prime }+A_{1}\psi^{B_{1}}$	(1)
Vanapalli et al. [12]	$\tau =c^{\prime }+\left(\sigma -u_{a}\right)\tan\varphi^{\prime }+\chi \psi \tan\varphi^{\prime }$	(2)
Öberg and Sallfors [25]	$\tau =c^{\prime }+\left(\sigma -u_{a}\right)\tan\varphi^{\prime }+S_{r}\psi \tan\varphi^{\prime }$	(3)
Xu and Sun [26]	$\tau =c^{\prime }+\left(\sigma -u_{a}\right)\tan\varphi^{\prime }+{m_{1}}^{\left(1-\xi_{1}\right)}\psi^{\xi_{1}}\tan\varphi^{\prime }$	(4)
Vilar [13]	$\tau =c^{\prime }+\left(\sigma -u_{a}\right)\tan\varphi^{\prime }+\frac{\psi }{\frac{1}{\tan\varphi^{\prime }}+(\frac{1}{c_{m}-c^{\prime }}-\frac{1}{\psi_{m}\tan\varphi^{\prime }})}$	(5)
Han and Vanapalli [14]	$\frac{\tau -\tau_{sat}}{\tau_{ref}-\tau_{sat}}=\frac{\psi }{\psi_{ref}}{\left(\frac{S_{r}}{S_{r-ref}}\right)}^{\xi_{2}}$	(6)

Note: $c^{\prime }$: effective cohesion; $\varphi^{\prime }$: effective friction angle; σ: normal stress; $u_{a}$: pore-air pressure; χ: effective stress parameter; $S_{r}$: degree of saturation; $c_{m}$ and $\psi_{m}$: measured maximum cohesion and the corresponding measured maximum suction; $\tau_{sat}$: shear strength under saturated condition; $\tau_{ref}$, $\psi_{ref}$ and $S_{r-ref}$: shear strength, suction and degree of saturation under a reference state; $A_{1}$, $B_{1}$, $m_{1}$, $\xi_{1}$ and $\xi_{2}$: model parameters. The SWRC was incorporated in Equations (2), (3) and (6).

(7) $$\tau =\tau_{sat}+f\left(\psi \right),\;f\left(0\right)=0$$

A factor of *χ*ψ was widely employed for describing the suction ψ effect on *τ* (e.g., Equation (2) in Vanapalli et al. [12], Equation (3) in Öberg and Sallfors [25], and Equation (6) in Han and Vanapalli [14], Table 2), where *χ* is the effective stress parameter. According to the findings of Han and Vanapalli [14], the use of *χ*ψ can upscale the pore-scale stress ψ to a macroscopic stress *χ*ψ, contributing to the unsaturated shear strength *τ*. This factor was modified in the present study, with consideration of the following two aspects: (a) *χ* was considered as the effective degree of saturation $S_{r}^{e}$, which was consistent with Alonso et al. [27] and Lu et al. [28]; (b) a power relationship between *τ* and ψ was reported by Abramento and Carvalho [11] and Xu and Sun [26] (Equations (1) and (4) in Table 2). In this circumstance, a factor of $S_{r}^{e}\psi^{B}$ was generated and, thus, Equation (8) was obtained below:

(8) $$\tau =\tau_{sat}+AS_{r}^{e}\psi^{B}$$

where *A* and *B* are constant parameters.

As conducted by Vilar [13] (Equation (5)) and Han and Vanapalli [14] (Equation (6)), one set of referenced experimental data was adopted in the modelling of unsaturated *τ*. Substituting a referenced shear strength *τ*_ref_, the corresponding effective degree of saturation $S_{r-ref}^{e}$ and suction ψ_ref_ in Equation (8) yields Equation (9):

(9) $$\tau_{ref}=\tau_{sat}+AS_{r-ref}^{e}\psi_{ref}^{B}$$

Equation (10) was derived by dividing Equation (8) with Equation (9), where the parameter *A* disappears:

(10) $$\frac{\tau -\tau_{sat}}{\tau_{ref}-\tau_{sat}}=\frac{S_{r}^{e}}{S_{r-ref}^{e}}{\left(\frac{\psi }{\psi_{ref}}\right)}^{B}$$

The empirical model by van Genuchten [21] was employed for the description of the SWRC:

(11) $$S_{r}^{e}=\frac{S_{r}-S_{r-r}}{1-S_{r-r}}={\left[\frac{1}{1+{\left(a\psi \right)}^{n}}\right]}^{m}$$

where the residual degree of saturation $S_{r-r}$ is taken as zero in the present study, *a* is a parameter with respect to the air-entry value, and *n* and *m* are constant parameters.

Combining Equation (10) with Equation (11), Equation (12) was deduced:

(12) $$\frac{\tau -\tau_{sat}}{\tau_{ref}-\tau_{sat}}={\left[\frac{1+{\left(a\psi_{ref}\right)}^{n}}{1+{\left(a\psi \right)}^{n}}\right]}^{m}{\left(\frac{\psi }{\psi_{ref}}\right)}^{B}$$

For the application of the proposed Equation (12), the information on (*i*) the shear strength under the saturated condition *τ*_sat_, (*ii*) a referenced shear strength *τ*_ref_ and the corresponding ψ_ref_ and (*iii*) the parameters *a*, *n* and *m* with respect to the SWRC were required. To verify the validity of the developed Equation (12), the present study, the study by Wang et al. [10] and another three previous studies (Rassam and Williams [29], Khalili et al. [30], Khalili and Zargarbashi [31] in Table 3) were selected.

Note that Duong et al. [3] were excluded, due to the fact that only two data points were obtained for a given *f*_v_ (Figure 5). For each study, the experimental results with varying *f*_v_ were separated into two groups. The first one was employed for the determination of model parameter *B* in Equation (12) (e.g., *f*_v_ = 0%, 10% and 35% in the present study and Wang et al. [10]; *f*_v_ = 37% in Rassam and Williams [29]; *f*_v_ = 16% in Khalili et al. [30]; *f*_v_ = 27% in Khalili and Zargarbashi [31]). On the other hand, the second group was employed for examining the performance of the developed Equation (12) with the parameter *B* previously determined (e.g., *f*_v_ = 20% and 45% in the present study and Wang et al. [10]; *f*_v_ = 49% in Rassam and Williams [29]; *f*_v_ = 25% in Khalili et al. [30]; *f*_v_ = 51% in Khalili and Zargarbashi [31]). 

**Figure 6:**
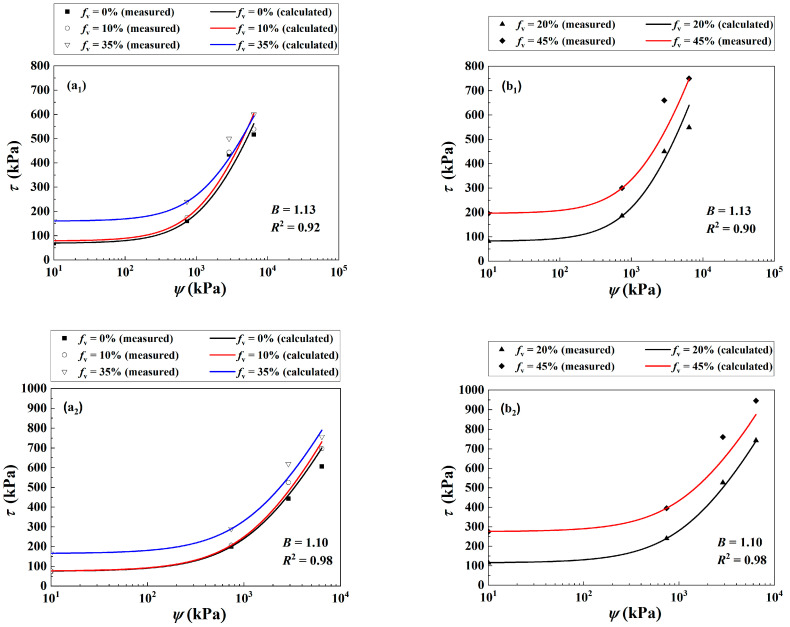

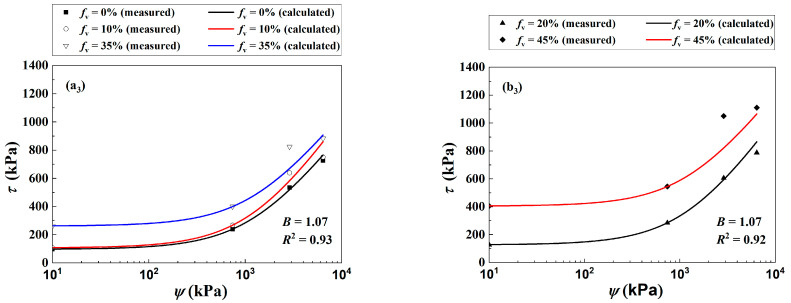
Comparisons between measured and calculated shear strength of the soil mixture with varying *f*_v_ and *ψ* under different *σ*_3_ values: (**a_1_**,**b_1_**) *σ*_3_ = 30 kPa, (**a_2_**,**b_2_**) *σ*_3_ = 60 kPa and (**a_3_**,**b_3_**) *σ*_3_ = 120 kPa (data from the present study and Wang et al. [10]).

The *τ* of the soil mixture with varying *f*_v_ = 0~45% was investigated under different *σ*_3_ = 30, 60 and 120 kPa in the present study (Figure 4) and Wang et al. [10]. The experimental results at *f*_v_ = 0%, 10% and 35% were employed for the determination of parameter *B* in Equation (12), while those at *f*_v_ = 20% and 45% were adopted to examine the performance of the proposed Equation (12). Figure 6(a_1_) compares the measured and the corresponding calculated *τ* of the mixture with *f*_v_ = 0%, 10% and 35% under *σ*_3_ = 30 kPa. Equation (12) provided satisfactory simulations with the coefficient of determination *R*^2^ = 0.92 using the parameter *B* = 1.13. Figure 6(b_1_) presents reasonably good agreement for the measured and calculated *τ* of the soil mixture at *f*_v_ = 20% and 45% with the previously determined *B* = 1.13 (*R*^2^ = 0.90). Similar observations were reported when *σ*_3_ increased to 60 kPa Figure 6(a_2_,b_2_) and 120 kPa Figure 6(a_3_,b_3_).

Equation (12) provided good simulations for the cases where *σ*_3_ = 60 and 120 kPa with parameter *B* = 1.10 and 1.07, respectively. The values of *R*^2^ for these two cases are shown in Figure 6(a_2_,b_2_) and Figure 6(a_3_,b_3_), which were at least larger than 0.92.

**Figure 7:**
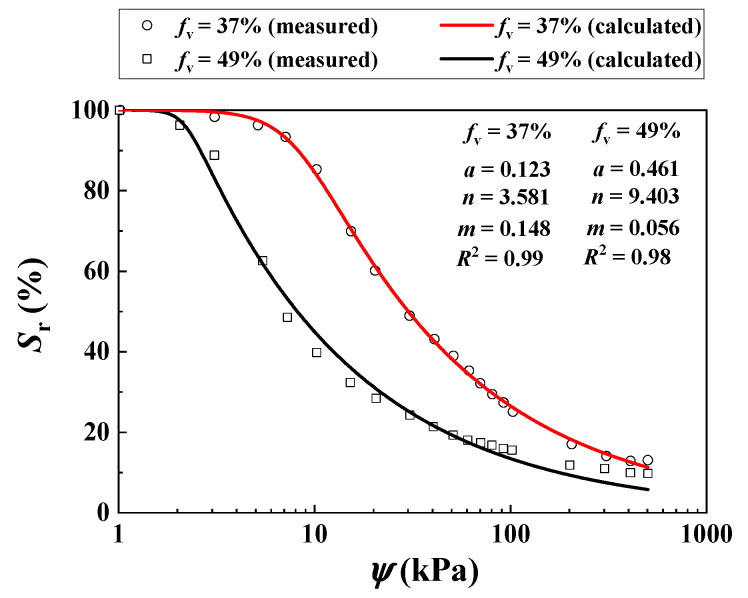
Measured and calculated SWRCs for the soil mixture with different *f*_v_ values (data from Rassam and Williams [29]).

Rassam and Williams [29] investigated the *τ* of two unsaturated tailing samples with different *f*_v_ = 37% and 49% under varying *σ*_3_ = 30, 125 and 250 kPa using suction-controlled triaxial tests. The values of ψ = 0, 20, 60 and 100 kPa were considered. Figure 7 depicts the SWRCs for *f*_v_ = 37% and 49%, respectively.

**Figure 8:**
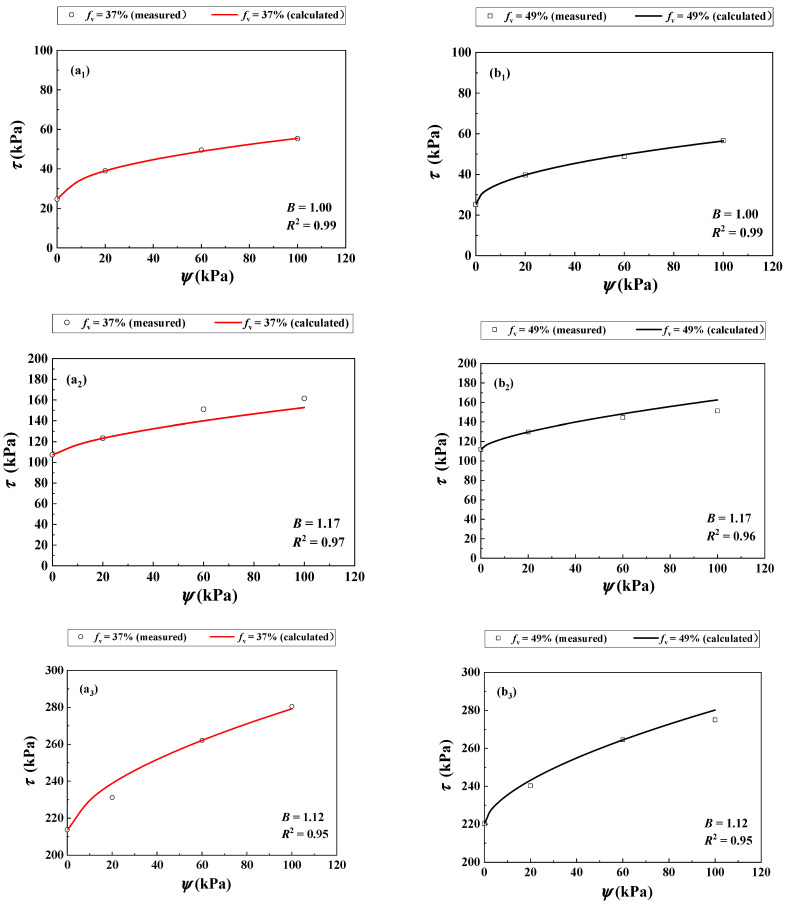
Comparisons between measured and calculated shear strength of the soil mixture with varying *f*_v_ and *ψ* under different *σ*_3_ values: (**a_1_**,**b_1_**) *σ*_3_ = 30 kPa, (**a_2_**,**b_2_**) *σ*_3_ = 125 kPa and (**a_3_**,**b_3_**) *σ*_3_ = 250 kPa (data from Rassam and Williams [29]).

Figure 8(a_1_) compares the measurements by Rassam and Williams [29] and the calculations using Equation (12) for *f*_v_ = 37% under *σ*_3_ = 30 kPa.

Equation (12) provided the calculated results using parameter *B* = 1.00. Figure 8(b_1_) compares the measured and calculated *τ* for *f*_v_ = 49% using Equation (12) with the previously determined *B* = 1.00. The comparison shows satisfactory agreement with *R*^2^ = 0.99. Similar observations were made for *σ*_3_ = 125 kPa Figure 8(a_2_,b_2_) and *σ*_3_ = 250 kPa Figure 8(a_3_,b_3_). Equation (12) provided satisfactory simulations for *σ*_3_ = 125 and 250 kPa with parameter *B* =1.17 and 1.12, respectively. The values of *R*^2^ for these two cases were larger than 0.95.

**Figure 9:**
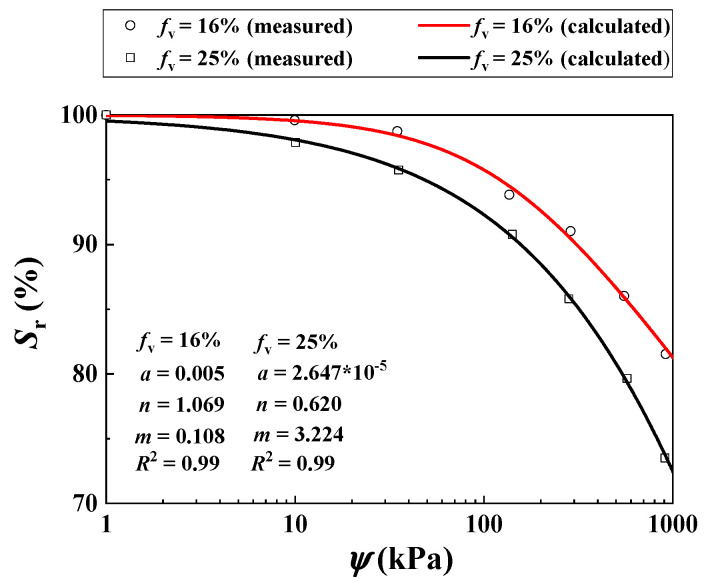
Measured and calculated SWRCs for the soil mixture with different *f*_v_ values (data from Khalili et al. [30]).

Khalili et al. [30] studied the *τ* of two unsaturated soil mixtures with varying *f*_v_ = 16% and 25% using suction-controlled triaxial tests. A constant *σ*_3_ = 200 kPa was adopted. The values of ψ = 0, 100, 200 and 400 were considered. When the *f*_v_ increased from 16% to 25%, the *ρ*_d_ of the mixture increased from 1.69 to 1.91 Mg/m^3^ (Table 3). Figure 9 compares the measurements and the calculations for the SWRCs for *f*_v_ = 16% and 25% using Equation (11).

**Figure 10:**
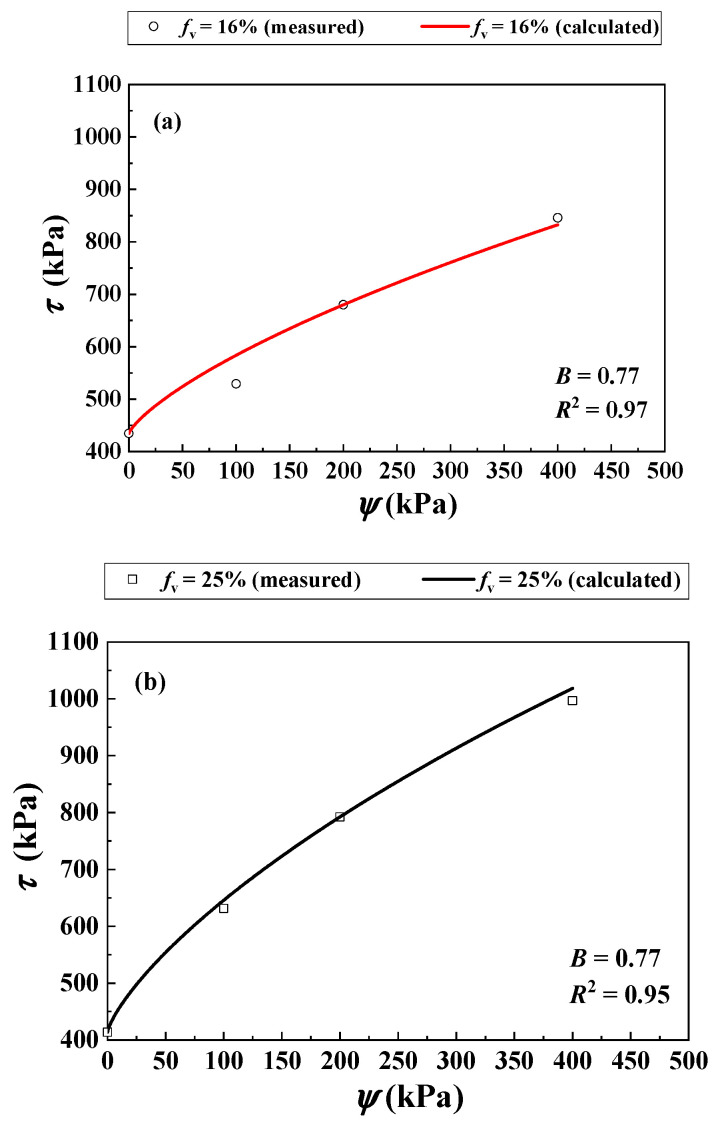
Comparisons between measured and calculated shear strength of the soil mixture with varying *f*_v_ and *ψ* under $\sigma_{3}$ = 200 kPa: (**a**) *f*_v_ = 16%; (**b**) *f*_v_ = 25% (data from Khalili et al. [30]).

Note that the parameters *a*, *n* and *m* with respect to the SWRC are presented in Table 3. Figure 10a compares the measurements by Khalili et al. [30] and the simulations using the developed Equation (12) for *f*_v_ = 16%. Equation (12) provided satisfactory simulations using the parameter *B* = 0.77 with *R*^2^ = 0.97.

Figure 10b shows the measured and predicted *τ* for *f*_v_ = 25% from Equation (12) using the aforementioned parameter *B* = 0.77. The comparison shows good agreement (*R*^2^ = 0.95).

**Figure 11:**
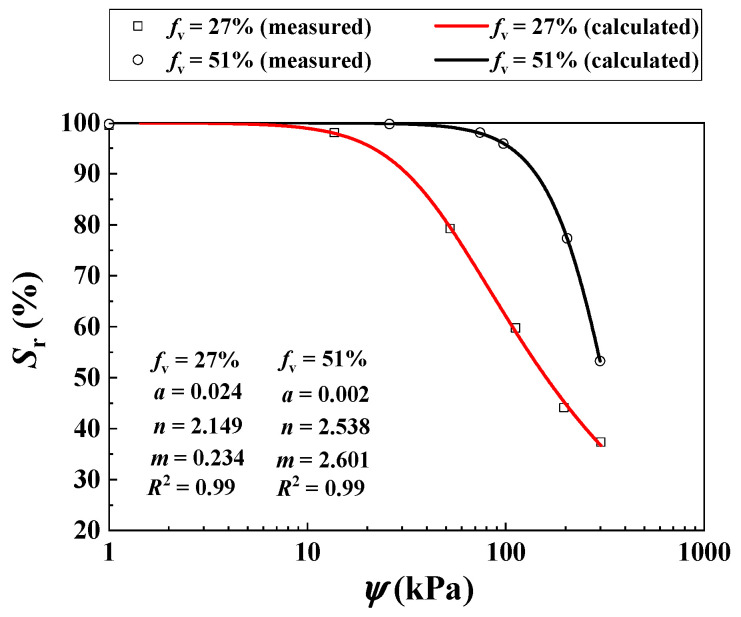
Measured and calculated SWRCs of the mixture with different *f*_v_ values (data from Khalili and Zargarbashi [31]).

Khalili and Zargarbashi [31] investigated the *τ* of two unsaturated soil mixtures with varying *f*_v_ = 27% and 51% using multi-stage shear tests. The *σ*_3_ was kept constant at 200 kPa. The values of ψ = 0, 30, 70, 100, 200 and 300 were considered for *f*_v_ = 27%, while those of ψ = 0, 15, 50, 110, 200 and 300 were considered for *f*_v_ = 51%. An increment of *f*_v_ from 27% to 51% induced an increasing *ρ*_d_ of the mixture from 1.53 to 1.63 Mg/m^3^ (Table 3). Figure 11 presents the SWRCs for *f*_v_ = 27% and 51%, which was fitted to Equation (11) (see the values of parameters *a*, *n*, *m* in Table 3).

**Figure 12:**
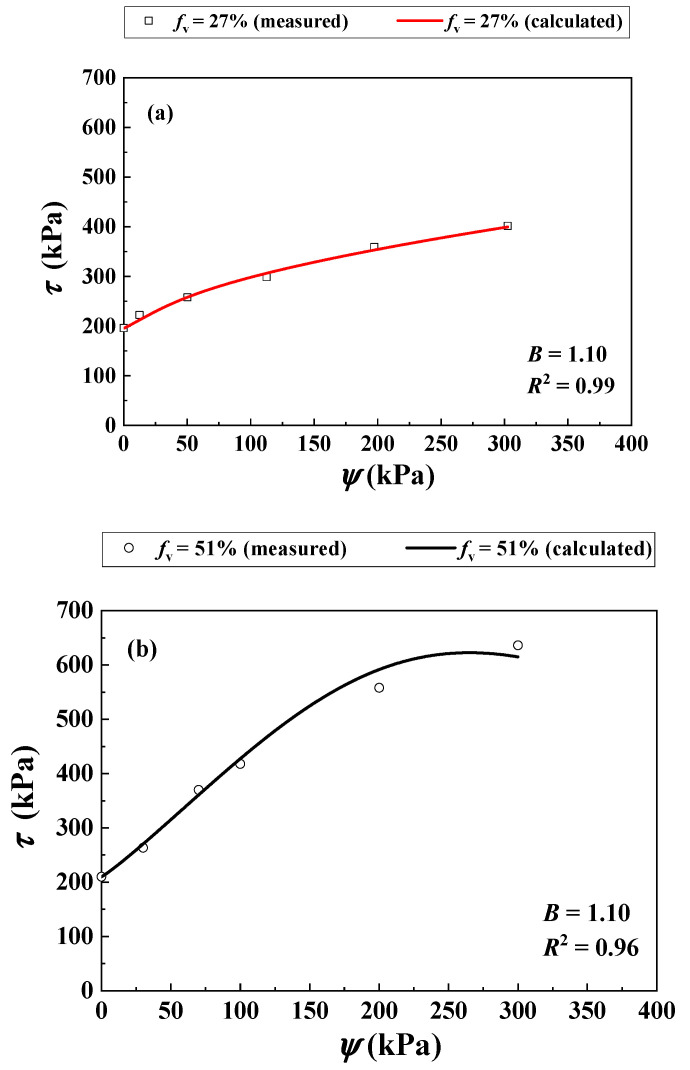
Comparisons between measured and calculated shear strength of the soil mixture with varying *f_v_* and *ψ* under $\sigma_{3}$ = 200 kPa: (**a**) *f*_v_ = 27%; (**b**) *f*_v_ = 51% (data from Khalili and Zargarbashi [31]).

Figure 12a compares the measurements by Khalili and Zargarbashi [30] and the predictions using the developed Equation (12) for *f*_v_ = 27%. Equation (12) provided a good description using the parameter *B* = 1.10 (*R*^2^ = 0.99).

Figure 12b presents satisfactory agreement between the measured and the calculated *τ* for *f*_v_ = 51% using Equation (12) with the aforementioned parameter *B* = 1.10 (*R*^2^ = 0.96).

**Figure 13:**
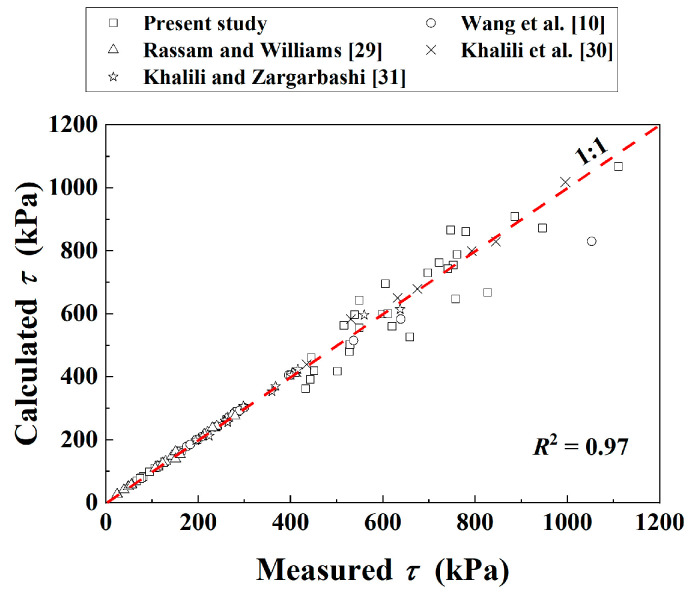
Comparisons between measured and calculated shear strength of the soil mixture in all studies.

Figure 13 compares the measured and the calculated *τ* of the soil mixture in all the studies. Good agreement was obtained (*R*^2^ = 0.97). The comparisons show the good performance of the developed Equation (12) in describing the *τ–*ψ relationship of the soil mixture with different *f*_v_.

## 5. Conclusions

Monotonic triaxial tests were fulfilled to investigate the combined influence of *f*_v_ and *w* on the *τ* of the soil mixture. Meanwhile, modelling of the *τ* of the soil mixture with varying *f*_v_ was conducted, which was examined using both the experimental results from the present study and those from previous studies. The results allow the following conclusions to be drawn.

It was found that in the present study, increasing the *f*_v_ led to an increase in the *τ* of the soil mixture under both saturation and unsaturation. Conversely, Duong et al. [3] reported that incrementally increasing the *f*_v_ induced an increment in the *τ* under saturation, but a decline under unsaturation. This was explained by the combined influence of coarse grain content *f*_v_ and suction ψ: in Duong et al. [3], the increment in *f*_v_ contributed to a decline in *ρ*_d–f_ and, thus, a decline in ψ. While the negative influence of the declining ψ outweighed the positive influence of the increasing *f*_v_, the *τ* of the soil mixture decreased. However, an unchanged *ρ*_d–f_ = 1.82 Mg/m^3^ was controlled in the present study. The incrementally increasing *f*_v_ contributed to an increment in the *τ* under both saturation and unsaturation, due to its only positive reinforcement influence.

Modelling of the *τ* of the mixture with various *f*_v_ was performed by incorporating the SWRC. The combined influence of the *ρ*_d–f_ and *w* on the *τ* was reflected by the influence of the ψ. This model was examined using the experimental results from both the present study and previous studies. Comparisons between the measurements and the predictions show the satisfactory performance of the developed model for the description of the *τ–*ψ relationship of the soil mixture with varying *f*_v_.

## Figures and Tables

**Table 1:** Cohesion and friction angle of the soil mixture with varying *f*_v_ and *w*_f_ values.

*W*_f_ (%)	*f*_v_ = 0%		*f*_v_ = 10%		*f*_v_ = 20%		*f*_v_ = 35%		*f*_v_ = 45%	
	*c*	*φ*	*c*	*φ*	*c*	*φ*	*c*	*φ*	*c*	*φ*
7	146	29	141	32	124	36	113	40	99	48
11	137	21	129	27	125	27	102	38	98	46
18	32	8	32	9	29	12	27	22	26	34

**Table 3:** Soil properties of the soil mixture in previous studies.

Reference	*f*_v_ (%)	Fine Soil Fraction					Soil Mixture		SWRC Fitted by van Genuchten [21] Model		
		*G*_s_	*w*_L_ (%)	*I*_p_ (%)	*w*_opt-f_ (%)	*ρ*_dmax-f_ (Mg/m^3^)	*ψ* (kPa)	*ρ*_d_ (Mg/m^3^)	*a*	*n*	*m*
Rassam and Williams [29]	37	N/A					0, 20, 60 and 100	N/A	0.123	3.581	0.149
	49								0.461	9.403	0.056
Khalili et al. [30]	16	N/A	33	12	N/A		0, 100, 200 and 400	1.69	0.005	1.069	0.108
	25							1.91	2.647 × 10^−5^	0.620	3.224
Khalili and Zargarbashi [31]	27	N/A					0, 30, 70, 100, 200 and 300	1.53	0.024	2.149	0.234
	51						0, 15, 50, 110, 200 and 300	1.63	0.002	2.538	2.601

## Data Availability

Not applicable.

## Notations

*c*	apparent cohesion
$c^{\prime }$	effective cohesion
$\varepsilon_{a}$	axial strain
$\varepsilon_{v}$	volumetric strain
*f*_v_	volumetric coarse grain content
*f*_v-cha_	characteristic volumetric coarse grain content
*G*_s_	specific gravity
*I*_p_	plasticity index
*ρ*_d_	dry density of sample
*ρ*_d–f_	dry density of fine soil
*ρ*_dmax-f_	maximum dry density of fine soil
*q*	deviator stress
*τ*	shear stress
*τ*_sat_	shear stress under saturated condition
*τ*_ref_	shear stress under referenced condition
*S*_r_	degree of saturation
*S*_r-r_	residual degree of saturation
$S_{r}^{e}$	effective degree of saturation
$S_{r-ref}^{e}$	effective degree of saturation under a referenced state
*w*	water content of soil mixture
*w*_f_	water content of fine soil
*w*_opt-f_	optimum water content of fine soil
*w*_L_	liquid limit
*χ*	effective stress parameter
$\sigma_{3}$	confining pressure
*φ*	friction angle
$\varphi^{\prime }$	effective friction angle
ψ	suction
ψ _ref_	suction under referenced state

## References

[1] Trinh V.N. (2011). Comportement Hydromecanique des Materiaux Constitutifs de Plateformes Ferroviaires Anciennes. Ph.D. Thesis.

[2] Trinh V.N., Tang A.M., Cui Y.J., Dupla J.C., Canou J., Calon N., Lambert L., Robinet A., Schoen O. (2012). Mechanical characterisation of the fouled ballast in ancient railway track substructure by large-scale triaxial tests. Soils Found..

[3] Duong T.V., Tang A.M., Cui Y.J., Trinh V.N., Dupla J.C., Calon N., Canou J., Robinet A. (2013). Effects of fines and water contents on the mechanical behavior of interlayer soil in ancient railway sub-structure. Soils Found..

[4] Qi S., Cui Y.J., Chen R.P., Wang H.L., Lamas-Lopez F., Aimedieu P., Dupla J.C., Canou J., Saussine G. (2020). Influence of grain size distribution of inclusions on the mechanical behaviours of track-bed materials. Géotechnique.

[5] Qi S., Cui Y.J., Dupla J.C., Chen R.P., Wang H.L., Su Y., Lamas-Lopez F., Canou J. (2020). Investigation of the parallel gradation method based on the response of track-bed materials under cyclic loadings. Transp. Geotech..

[6] Wan Z., Bian X., Li S., Chen Y., Cui Y. (2020). Remediation of mud pumping in ballastless high-speed railway using polyurethane chemical injection. Constr. Build. Mater..

[7] Bian X., Wan Z., Zhao C., Cui Y., Chen Y. (2021). Mud pumping in the roadbed of ballastless high-speed railway. Géotechnique.

[8] Vallejo L.E. (2001). Interpretation of the limits in shear strength in binary granular mixtures. Can. Geotech. J..

[9] Seif El Dine B., Dupla J.C., Frank R., Canou J., Kazan Y. (2010). Mechanical characterization of matrix coarse-grained soils with a large-sized triaxial device. Can. Geotech. J..

[10] Wang H.L., Cui Y.J., Lamas-Lopez F., Calon N., Saussine G., Dupla J.C., Canou J., Aimedieu P., Chen R.P. (2018). Investigation on the mechanical behavior of track-bed materials at various contents of coarse grains. Constr. Build. Mater..

[11] Abramento M., Carvalho C.S. (1989). Geotechnical parameters for the study of natural slopes instabilization at ‘Serra do Mar’, Brazil. Congrès Intrnational de Mécanique des Sols et des Travaux de Fondations.

[12] Vanapalli S.K., Fredlund D.G., Pufahl D.E., Clifton A.W. (1996). Model for the prediction of shear strength with respect to soil suction. Can. Geotech. J..

[13] Vilar O.M. (2006). A simplified procedure to estimate the shear strength envelope of unsaturated soils. Can. Geotech. J..

[14] Han Z., Vanapalli S.K. (2016). Stiffness and shear strength of unsaturated soils in relation to soil-water characteristic curve. Géotechnique.

[15] Wang H.L., Cui Y.J., Lamas-Lopez F., Dupla J.C., Canou J., Calon N., Saussine G., Aimedieu P., Chen R.P. (2018). Permanent deformation of track-bed materials at various inclusion contents under large number of loading cycles. J. Geotech. Geoenviron. Eng..

[16] Su Y., Cui Y.J., Dupla J.C., Canou J., Qi S. (2021). Developing a Sample Preparation Approach to Study the Mechanical Behavior of Unsaturated Fine/Coarse Soil Mixture. Geotech. Test. J..

[17] Han Z., Vanapalli S.K. (2016). Relationship between resilient modulus and suction for compacted subgrade soils. Eng. Geol..

[18] Yang S.R., Huang W.H., Tai Y.T. (2005). Variation of resilient modulus with soil suction for compacted subgrade soils. Transp. Res. Rec..

[19] Yang S.R., Lin H.D., Kung J.H., Huang W.H. (2008). Suction-controlled laboratory test on resilient modulus of unsaturated compacted subgrade soils. J. Geotech. Geoenviron. Eng..

[20] Su Y., Cui Y.J., Dupla J.C., Canou J. (2022). Soil-water retention behaviour of fine/coarse soil mixture with varying coarse grain contents and fine soil dry densities. Can. Geotech. J..

[21] Van Genuchten M.T. (1980). A closed-form equation for predicting the hydraulic conductivity of unsaturated soils. Soil Sci. Soc. Am. J..

[22] (2011). Standard Test Method for Consolidated Drained Triaxial Compression Test for Soils (Superseded).

[23] Delage P., Audiguier M., Cui Y.J., Howat M.D. (1996). Microstructure of a compacted silt. Can. Geotech. J..

[24] Ng C.W.W., Baghbanrezvan S., Sadeghi H., Zhou C., Jafarzadeh F. (2017). Effect of specimen preparation techniques on dynamic properties of unsaturated fine-grained soil at high suctions. Can. Geotech. J..

[25] Öberg A.L., Sallfors G. (1997). Determination of shear strength parameters of unsaturated silts and sands based on the water retention curve. Geotech. Test. J..

[26] Xu Y.F., Sun D.A. (2002). A fractal model for soil pores and its application to determination of water permeability. Phys. A Stat. Mech. Appl..

[27] Alonso E.E., Pereira J.M., Vaunat J., Olivella S. (2010). A microstructurally based effective stress for unsaturated soils. Géotechnique.

[28] Lu N., Godt J.W., Wu D.T. (2010). A closed-form equation for effective stress in unsaturated soil. Water Resour. Res..

[29] Rassam D.W., Williams D.J. (1999). A relationship describing the shear strength of unsaturated soils. Can. Geotech. J..

[30] Khalili N.G.F.A., Geiser F., Blight G.E. (2004). Effective stress in unsaturated soils: Review with new evidence. Int. J. Geomech..

[31] Khalili N., Zargarbashi S. (2010). Influence of hydraulic hysteresis on effective stress in unsaturated soils. Géotechnique.

